# Cutaneous Complications in Type 1 Diabetes: Influence of Smoking

**DOI:** 10.7759/cureus.106513

**Published:** 2026-04-06

**Authors:** Basil Afzal, Syed Arsalan Ahmed, Maaz Ali, Amira Younes, Bakhtawar Farooq, Lakshmi Balakrishnan, Adnan Anwar, Atif A Hashmi

**Affiliations:** 1 Internal Medicine, Calderdale and Huddersfield NHS Foundation Trust, Halifax, GBR; 2 Internal Medicine, Calderdale Royal Hospital, Halifax, GBR; 3 Emergency Medicine, Calderdale and Huddersfield NHS Foundation Trust, Halifax, GBR; 4 Physiology, Hamdard College of Medicine and Dentistry, Karachi, PAK; 5 Internal Medicine, Sindh Governemnt Hospital, Malir, PAK; 6 Pathology, Indus Hospital and Health Network, Karachi, PAK

**Keywords:** dermatological manifestations, diabetic complications, smoking, type 1 diabetes, xerosis

## Abstract

Objective: Type 1 diabetes mellitus (T1DM) often presents with various skin complications, which may be worsened by smoking due to its vascular and metabolic effects. This study aimed to evaluate the influence of smoking on cutaneous manifestations and compare the demographic and clinical characteristics of smokers and non-smokers with T1DM.

Methodology: This cross-sectional study was performed at multiple secondary care hospitals and primary care centers. The duration of the study was about six months from November 1, 2024, to April 30, 2025. This study included 220 patients with confirmed T1DM, categorized into two groups based on smoking status: Group A included current smokers (n = 110), while Group B comprised non-smokers (n = 110). Data were collected through clinical examinations and structured questionnaires, including demographic details, lifestyle factors, physiological parameters, and dermatological findings. Statistical analysis was applied using the chi-square test and a Mann-Whitney test, with p < 0.05 reflecting statistical significance.

Results: The findings of the study showed that the smokers had a significantly lower mean height (66.17 ± 10.71 inches), BMI (21.18 ± 5.61 kg/m²), respiratory rate (17.34 ± 5.89 cycles/min), and oral glucose tolerance test (OGTT) values (223.26 ± 75.21 mg/dL) compared to non-smokers (p < 0.05). Most smokers were male, 81 (73.6%), whereas the majority of non-smokers were female, 85 (77.3%) (p < 0.001). Regarding cutaneous manifestations, xerosis fissured skin (59, 53.6%) and bullae (33, 30.0%) were significantly more prevalent in smokers, while ichthyosis (26, 23.6%) and diabetic rubeosis (17, 15.5%) were more frequent among non-smokers (p < 0.05).

Conclusion: This study concluded that smoking significantly affects the pattern of skin manifestations in patients with type 1 diabetes mellitus. Smokers showed a higher prevalence of xerosis, acanthosis nigricans, and bullae, while non-smokers more often exhibited ichthyosis and diabetic rubeosis.

## Introduction

Type 1 diabetes mellitus (T1DM) is the most common type of diabetes seen in children and adolescents [1], though it can also manifest during adulthood [2,3]. It is now recognized that T1DM typically begins with the appearance of islet cell autoantibodies, often several years before clinical symptoms develop [4]. However, studies have shown that in certain South Asian populations [5], these commonly used autoantibodies are less frequently detected compared to European populations, even among individuals with low C-peptide levels.

The World Health Organization defines diabetes mellitus as a metabolic disorder with multiple causes, marked by persistent hyperglycemia and disturbances in carbohydrate, protein, and fat metabolism due to impaired insulin secretion, insulin action, or both. Approximately 79% of individuals with diabetes experience skin manifestations at some stage of their disease [6]. According to recent estimates, in 2021, approximately 536.6 million adults aged 20-79 years (10.5%) worldwide were living with diabetes, a number projected to rise to over 780 million (12.2%) by 2045. The prevalence is notably higher in urban areas (12.1%) and high-income countries compared to rural (8.3%) and low-income regions [7,8].

The prevalence of diabetes is increasing rapidly, particularly in low- and middle-income countries. Smokers, whether diabetic or not, tend to exhibit higher insulin resistance, resulting in poor glycemic control [9]. In an observational study by Peng et al. involving over 25,000 individuals with diabetes, current smokers had a 1.5 times higher risk of inadequate glycemic control, defined as HbA1c ≥ 53 mmol/mol (7.0%), as compared to non-smokers [10]. These findings indicate a link between smoking and difficulty in achieving glycemic targets. However, the impact of smoking on glycemic variability remains less thoroughly explored.

Approximately 30% of diabetic patients experience cutaneous manifestations at some stage of their illness. Although the overall frequency of skin disorders is similar in type I and type II diabetes, patients with type II diabetes tend to develop more infectious skin conditions, while those with T1DM are more prone to autoimmune-related lesions [8]. These dermatological changes usually appear after the onset of diabetes but may occasionally precede its diagnosis by several years. Diabetes affects multiple organ systems, including the skin, eyes, kidneys, blood vessels, and nerves, through mechanisms such as abnormal carbohydrate metabolism, atherosclerosis, microangiopathy, neuronal degeneration, and impaired immune function [11]. Several classifications of diabetic skin disorders have been described in the literature. According to one study, no skin condition is entirely specific to diabetes, although three dermatoses are seen more frequently in diabetic patients than in non-diabetics [12]. In one study, cutaneous infections were the most prevalent skin manifestation (45.7%), followed by inflammatory skin diseases (27.7%) and xerosis (6.4%) [13].

Diabetic foot syndrome (DFS) refers to a combination of neuropathic and vascular complications affecting the feet of individuals with diabetes. Although largely preventable, DFS remains a major contributor to morbidity, mortality, hospital admissions, and reduced quality of life among diabetic patients. The incidence and prevalence of DFS are estimated at 1-4% and 4-10%, respectively [14]. Scleroderma-like skin changes represent a distinct yet often overlooked manifestation commonly seen in diabetic individuals, occurring in about 10-50% of cases [15]. Xerosis, or abnormally dry skin, is among the most frequent dermatological manifestations in diabetes, reported in up to 40% of patients [16]. It is characterized by roughness, scaling, and fissuring, most commonly affecting the feet. Studies have also noted that obese diabetic patients tend to experience more pronounced hypohidrosis (reduced sweating) of the feet, worsening the dryness [17].

Therefore, the objective of this study was to assess and compare the frequency and pattern of cutaneous complications among smokers and non-smokers with T1DM in the Pakistani population.

## Materials and methods

This cross-sectional study was performed at multiple secondary care hospitals and primary care centers using a non-probability convenient sampling technique. The ethical approval of the study was obtained from the Sindh Govt. Hospital, Malir, Karachi, Pakistan (SGH/2-A(Landhi)/1571). The duration of the study was about six months, from November 1, 2024, to April 30, 2025. Using Open Epi software for sample size estimation, the prevalence of skin-related complications in patients with T1DM was taken as 79.2%, based on a previously published study [6]. The expected sample size was 253 patients. The study included 220 patients diagnosed with T1DM aged 18 years and above of both genders, who were divided into two groups based on their smoking status. Group A included current smokers (n = 110), while Group B comprised non-smokers (n = 110) with no history of tobacco use. Patients were excluded if they had type 2 diabetes mellitus, gestational diabetes, or any other systemic condition known to independently influence skin health, including thyroid disorders, chronic renal disease, hepatic dysfunction, or autoimmune dermatological diseases (e.g., lupus erythematosus, pemphigus vulgaris, or psoriasis). In addition, individuals with pre-existing dermatological conditions unrelated to diabetes that could confound skin assessment, those receiving long-term systemic corticosteroid therapy, immunosuppressive agents, or other medications known to alter skin physiology, and patients with a history of recent acute systemic illness or hospitalization were excluded. To strengthen internal validity, potential confounding variables such as age, sex, body mass index, smoking status, and other relevant comorbidities were documented and controlled for during statistical analysis where applicable. This structured approach was adopted to ensure methodological rigor, reduce bias, and improve the reproducibility of the findings.

Informed consent was obtained from all participants before data collection. Comprehensive clinical assessments were conducted to evaluate glycemic control and identify skin complications associated with diabetes. Glycemic status was determined using glycosylated hemoglobin (HbA1c) levels, which indicate long-term blood glucose regulation, and an oral glucose tolerance test. Participants were instructed to undergo an overnight fast of 8-12 hours, during which only water was allowed, prior to testing. A standard oral glucose tolerance test (OGTT) was then performed by administering 75 g of anhydrous glucose dissolved in 250-300 mL of water, which was consumed within 5 minutes. Plasma glucose levels were measured and interpreted according to established diagnostic criteria, with impaired glucose tolerance (IGT) defined as a two-hour post-load plasma glucose level of 140-199 mg/dL (7.8-11.0 mmol/L), while fasting plasma glucose remained below 126 mg/dL. Cardiovascular health was assessed through physical measurements such as blood pressure and heart rate.

Data collection involved a structured questionnaire and detailed clinical examination covering demographic characteristics like age, lifestyle factors, clinical parameters, and dermatological findings. Anthropometric measurements (height, weight, and BMI), physiological parameters (blood pressure, respiratory rate, and heart rate), and biochemical assessments, such as an oral glucose tolerance test, were recorded. Dyslipidemia was diagnosed based on lipid profile analysis. All participants underwent thorough dermatological examinations conducted by trained clinicians under standardized conditions to identify skin conditions commonly linked to T1DM. Findings were documented using a predefined checklist and standardized diagnostic criteria. The key dermatological manifestations assessed included xerosis, ichthyosis, diabetic rubeosis, acanthosis nigricans, callosities, and bullae.

Data was analyzed using IBM Corp. Released 2014. IBM SPSS Statistics for Windows, Version 20. Armonk, NY: IBM Corp. The demographic information and dermatological manifestations associated with T1DM were represented as frequencies and percentages. Quantitative variables were presented as means and standard deviations. A chi-square test was applied to detect the association of dermatological features in T1DM. Moreover, a Mann-Whitney test was applied to compare variables between two independent groups. A p-value of < 0.05 was considered statistically significant.

## Results

Table 1 presents the demographic and clinical characteristics of patients with T1DM based on smoking status. The mean age of smokers (Group A) was 52.00 ± 14.32 years, while non-smokers (Group B) had a mean age of 55.00 ± 16.65 years, showing no significant difference (p = 0.261). The mean height of Group A (66.17 ± 10.71 inches) was significantly lower than that of Group B (70.90 ± 7.78 inches) (p = 0.001). The mean weight was slightly higher in non-smokers (67.00 ± 12.93 kg) compared to smokers (64.70 ± 15.95 kg), though the difference was statistically insignificant (p = 0.071). Body mass index (BMI) was significantly lower among smokers (21.18 ± 5.61 kg/m²) compared to non-smokers (23.92 ± 7.34 kg/m²) (p = 0.006). Similarly, respiratory rate was significantly lower in smokers (17.34 ± 5.89 cycles/min) than in non-smokers (19.29 ± 5.71 cycles/min) (p = 0.003). Moreover, insignificant differences were observed between the two groups in systolic blood pressure (154.91 ± 46.22 mmHg vs. 158.63 ± 40.73 mmHg, p = 0.586), duration of hypertension (3.32 ± 3.82 years vs. 3.45 ± 5.47 years, p = 0.313), or heart rate (82.97 ± 12.55 beats/min vs. 84.78 ± 10.71 beats/min, p = 0.147). However, the mean oral glucose tolerance test (OGTT) value was significantly lower in smokers (223.26 ± 75.21 mg/dL) compared to non-smokers (247.10 ± 68.95 mg/dL) (p = 0.004).

**Table 1:** The demographic details of patients with T1DM based on duration of diabetes (n=220) Group A represents smokers, while Group B represents non-smokers. Values are expressed as mean ± SD; p-values and U-statistics were calculated using the Mann-Whitney test. *p-value significant as <0.05

Variables	Group A (n=110) Mean±SD	Group B (n=110) Mean±SD	Mann–Whitney U	p-value
Age (years)	52.00±14.32	55.00±16.65	5520.000	0.261
Height (Inch)	66.17±10.71	70.90±7.78	4497.000	0.001*
Weight (kg)	64.70±15.95	67.00±12.93	5201.000	0.071
BMI (kg/m^2^)	21.18±5.61	23.92±7.34	4747.500	0.006*
Respiratory Rate (cycles/min)	17.34±5.89	19.29±5.71	4635.500	0.003
Systolic Blood pressure (mmHg)	154.91±46.22	158.63±40.73	5795.500	0.586
Duration of hypertension (Years)	3.32±3.82	3.45±5.47	5587.000	0.313
Heart rate (beats/min)	82.97±12.55	84.78±10.71	5368.500	0.147
Oral glucose tolerance test (OGTT) (mg/dL)	223.26±75.21	247.1±68.95	4698.500	0.004*

Table 2 compares the demographic, lifestyle, and clinical characteristics of smokers and non-smokers with T1DM. Age distribution showed no significant difference between the two groups (p = 0.145), though a higher proportion of non-smokers were aged above 50 years (60, 54.5%) compared to smokers (54, 49.1%). Gender distribution differed significantly, with a majority of smokers being male (81, 73.6%) and most non-smokers being female (85, 77.3%) (p < 0.001). Body mass index (BMI) classification also revealed a significant difference (p < 0.001), as more smokers were underweight (60, 54.5%), while non-smokers had a higher prevalence of overweight individuals (46, 41.8%). Socioeconomic status was significantly associated with smoking status (p = 0.005), with a greater proportion of smokers belonging to the middle-income group (60, 54.5%), while more non-smokers were in the low-income category (34, 30.9%). The history of hypertension did not differ significantly between groups (p = 0.497). However, dyslipidemia was significantly more common among smokers (84, 76.4%) compared to non-smokers (58, 52.7%) (p < 0.001). Similarly, a history of depression was more prevalent among smokers (35, 31.8%) than non-smokers (15, 13.6%) (p = 0.001). Physical activity differed markedly between the groups (p < 0.001), as only 17 (15.5%) of smokers reported being physically active compared to 49 (44.5%) of non-smokers.

**Table 2:** Comparison of demographic, lifestyle, and clinical characteristics based on smoking status *p-value is significant as < 0.05. Group A: Smokers (n = 110) Group B: Non-smokers (n = 110) Values are presented as n (%). Associations were assessed using the Pearson chi-square test. Effect size was estimated using Cramer’s V. A p-value < 0.05 was considered statistically significant.

Variables		Group A (n=110)	Group B (n=110)	Pearson Chi-square	Cramer’s V	p-value
Age groups (years)	18-30	3(2.7%)	8(7.3%)	3.862	0.132	0.145
	31-50	53(48.2%)	42(38.2%)			
	Above 50	54(49.1%)	60(54.5%)			
Gender	Male	81(73.6%)	25(22.7%)	57.094	0.509	<0.001*
	Female	29(26.4%)	85(77.3%)			
BMI (kg/m^2^)	<18.5	60(54.5%)	31(28.2%)	18.122	0.287	<0.001*
	18.6- 24.9	28(25.5%)	33(30.0%)			
	>25.0	22(20.0%)	46(41.8%)			
Socioeconomic Status	Low	15(13.6%)	34(30.9%)	10.558	0.219	0.005*
	Middle	60(54.5%)	42(38.2%)			
	High	35(31.8%)	34(30.9%)			
History of Hypertension	Yes	59(53.6%)	64(58.2%)	0.461	0.046	0.497
	No	51(46.4%)	46(41.8%)			
Dyslipidemia	Yes	84(76.4%)	58(52.7%)	13.427	0.247	<0.001*
	No	26(23.6%)	52(47.3%)			
History of Depression	Yes	35(31.8%)	15(13.6%)	10.353	0.217	0.001*
	No	75(68.2%)	95(86.4%)			
Physical activity	Yes	17(15.5%)	49(44.5%)	22.165	0.317	<0.001*
	No	93(84.5%)	61(55.5%)			

Table 3 presents the dermatological symptoms observed among Group A and Group B with T1DM. Xerosis-fissured skin was significantly more prevalent among smokers, with 59 (53.6%) affected compared to 42 (38.2%) non-smokers (p = 0.021). Ichthyosis showed a highly significant difference (p < 0.001), occurring in only 4 (3.6%) smokers but in 26 (23.6%) non-smokers. Similarly, diabetic rubeosis was significantly more frequent among non-smokers, affecting 17 (15.5%) compared to just 2 (1.8%) smokers (p < 0.001). Acanthosis nigricans was more common in smokers, 46 (41.8%), than in non-smokers, 33 (30.0%), though this difference was not statistically significant (p = 0.068). Callosities were rare in both groups, present in 1 (0.9%) smoker and 3 (2.7%) non-smokers (p = 0.313). Bulla formation was significantly more common among smokers (33, 30.0%) compared to non-smokers (18, 16.4%) (p = 0.017).

**Table 3:** The dermatological symptoms in T1DM with smoking status *p-value significant as < 0.05. Group A: Smokers (n = 110) Group B: Non-smokers (n = 110) Values are presented as n (%). Associations were assessed using the Pearson chi-square test. Effect size was estimated using Cramer’s V. A p-value of < 0.05 was considered statistically significant.

Variables		Group A (n=110)	Group B (n=110)	Pearson Chi-square	Cramer’s V	p-value
Xerosis fissured skin	Yes	59(53.6%)	42(38.2%)	5.290	0.155	0.021*
	No	51(46.4%)	68(61.8%)			
Ichthyosis	Yes	4(3.6%)	26(23.6%)	18.681	0.291	<0.001*
	No	106(96.4%)	84(76.4%)			
Diabetic rubeosis	Yes	2(1.8%)	17(15.5%)	12.962	0.243	<0.001*
	No	108(98.2%)	93(84.5%)			
Acanthosis nigricans	Yes	46(41.8%)	33(30.0%)	3.338	0.123	0.068
	No	64(58.2%)	77(70.0%)			
Callosities	Yes	1(0.9%)	3(2.7%)	1.019	0.068	0.313
	No	109(99.1%)	107(97.3%)			
Bulla	Yes	33(30.0%)	18(16.4%)	5.743	0.162	0.017*
	No	77 (70.0%)	92(83.6%)			

## Discussion

The present study evaluated the impact of smoking on the prevalence and pattern of cutaneous complications in patients with T1DM. Specifically, it aims to compare the frequency and types of dermatological manifestations between smokers and non-smokers with T1DM. By identifying the influence of smoking on skin health in diabetic individuals, the study seeks to highlight smoking as a potential modifiable risk factor that exacerbates dermatological complications and compromises overall disease management in the Pakistani population.

One of the observational studies was carried out involving 200 patients diagnosed with diabetes who presented with various cutaneous manifestations. Type 2 diabetes was the predominant form among the participants. Most of the patients were female, and the majority belonged to the 46-55 years age group. Infections represented the most frequent skin manifestations, with fungal infections being the most prevalent. Among these, candidiasis and dermatophytosis were the most commonly observed types. Xerosis was identified in 18 patients, predominantly those suffering from diabetic neuropathy. Pruritus was another frequent finding, particularly noted in individuals with poorly controlled or early-stage diabetes [18]. In contrast, the present study on T1DM revealed a somewhat different pattern. Although age distribution showed no significant difference between smokers and non-smokers, males predominated among smokers, while females were more common in the non-smoking group. Xerosis is significantly higher among smokers, especially those with diabetic neuropathy. The present study found xerosis, acanthosis nigricans, and bullae to be more frequent among smokers, while ichthyosis and diabetic rubeosis were significantly more common among non-smokers. These findings indicate that smoking may alter the pattern of cutaneous manifestations in diabetes, with a greater prevalence of xerotic and bullous changes rather than infectious lesions.

Another study identified the most common cutaneous manifestations associated with diabetes. Among 103 patients examined, the predominant findings included diabetic foot (20%), bacterial infections (35%), fungal infections, and xerosis (45%). These manifestations were most frequent in individuals over 50 years of age, with a mean age of 63.3 years, and a higher prevalence among females (60 females, 43 males; F:M = 1.4:1). The most frequently observed diabetes-related lesions were diabetic foot (19 cases), followed by necrobiosis lipoidica (seven cases), bullosis diabeticorum (six cases), and acanthosis nigricans (five cases) [19]. Comparable gender distribution was also seen in the study by Timshina et al., which reported a female-to-male ratio of 1.21:1 [20]. Similarly, a study by Trihan et al. reported comparable findings, with necrobiosis lipoidica observed in five patients, acanthosis nigricans in four patients, and bullosis diabeticorum in three patients [21]. These findings were inconsistent with the present study, which reported that xerosis was significantly higher among smokers, while ichthyosis and diabetic rubeosis were more prevalent among non-smokers. Unlike earlier studies with a female predominance, the present study reported a higher proportion of males among smokers and females among non-smokers. Moreover, smoking was associated with a higher incidence of bullous lesions and acanthosis nigricans, suggesting that smoking may exacerbate certain non-infectious cutaneous complications in diabetic patients, in contrast to the infection-dominated profiles seen in previous research.

A study conducted by Jensus et al. included 49 smokers and 320 non-smokers. The results demonstrated that smokers had a 4.7-fold higher risk (95% CI: 1.5-15.4) of failing to achieve glycemic targets compared to non-smokers. Continuous glucose monitoring (CGM) revealed that smokers spent more time in hyperglycemia, had reduced time within the target glucose range, experienced more frequent hypoglycemic episodes (particularly very low interstitial glucose levels), and showed greater glucose fluctuations. The study concluded that smoking is associated with poor glycemic control and increased glycemic variability in individuals with T1DM, with an especially higher risk of morning hypoglycemia. Baseline characteristics from the same study showed no significant differences between smokers and non-smokers except for BMI, which was lower among smokers [22], an observation consistent with existing literature [23]. Lower BMI has been linked to greater glycemic variability and an increased risk of severe hypoglycemia [24,25]. Furthermore, Lohse et al. reported that heavy smoking correlates with unhealthy lifestyle habits, such as reduced consumption of fruits and vegetables and higher alcohol intake, the latter contributing to an elevated risk of hypoglycemia [26]. In comparison with the findings of Jensus et al. [22] and Lohse et al. [26], the present study similarly demonstrated a significant association between smoking and lower BMI among patients with T1DM. A pattern was also observed in the present study, where smokers showed a significantly lower mean BMI (21.18 ± 5.61 kg/m²) than non-smokers (23.92 ± 7.34 kg/m²; p = 0.006). Additionally, consistent with Lohse et al., who linked smoking with unhealthy lifestyle habits and reduced physical activity [26], the present study found a markedly lower proportion of physically active individuals among smokers (15.5%) compared to non-smokers (44.5%) (p < 0.001). These findings collectively support the notion that smoking in T1DM is associated with poorer metabolic and lifestyle profiles, characterized by lower BMI and decreased physical activity levels, both of which may contribute to suboptimal glycemic control and increased risk of complications. 

In one of our previous studies, we studied the association of the duration of type 2 DM with cutaneous manifestations. We found that certain cutaneous manifestations, such as acanthosis nigricans, callosities, and bullae, were significantly associated with longer duration of type 2 DM [27].

This study was limited by its cross-sectional design, which restricts the ability to establish causal relationships between smoking and dermatological manifestations in T1DM. Additionally, smoking history was based on self-reported data, introducing potential recall bias. Other confounding factors, such as duration and intensity of smoking, glycemic control, and comorbidities, were not fully accounted for. It is recommended that patients with T1DM be regularly screened for dermatological manifestations, especially those who smoke. Establishing consensus-based guidelines would help ensure early detection, risk stratification, and timely intervention, especially in high-risk groups like smokers. Smoking cessation programs should be integrated into diabetic care to reduce the risk of skin complications. Further longitudinal and multicenter studies are suggested to better understand the causal relationship between smoking and dermatological changes in T1DM.

## Conclusions

This study concluded that smoking status influences the pattern of dermatological manifestations in patients with type 1 diabetes mellitus. Smokers exhibited a higher prevalence of xerosis, fissured skin, acanthosis nigricans, and bulla, whereas non-smokers showed more frequent ichthyosis and diabetic rubeosis. These results suggest that smoking may exacerbate certain skin complications in T1DM, highlighting the importance of smoking cessation and regular dermatological evaluation in diabetic care.
